# A novel artificial tissue simulator for endoscopic submucosal resection training – a pilot study

**DOI:** 10.1186/s12876-016-0529-x

**Published:** 2016-09-10

**Authors:** Ming-Jen Chen, Horng-Yuan Wang, Chen-Wang Chang, Ching-Chung Lin, Chih-Jen Chen, Cheng-Hsin Chu, Tsang-En Wang, Shou-Chuan Shih

**Affiliations:** 1 Division of Gastroenterology, Department of Internal Medicine, MacKay Memorial Hospital, Taipei Campus, No. 92, Sec. 2, Chungshan North Road, Taipei, Taiwan; 2 MacKay Junior College of Medicine, Nursing and Management, Taipei, Taiwan; 3 Clinical Skills Training Center, Department of Medical Education, MacKay Memorial Hospital, Taipei Campus, Taipei, Taiwan; 4 MacKay Medical College, New Taipei City, Taiwan

**Keywords:** Simulation, Hands-on training, Endoscopy, Endoscopic submucosa dissection, Artificial tissue

## Abstract

**Background:**

We developed a novel artificial simulator for endoscopic submucosal dissection (ESD) as a bridge between instructional videos and animal tissue training and aimed to evaluate the feasibility of using an artificial tissue model in ESD training.

**Methods:**

Eight gastroenterology fellows from one medical center were enrolled in this ESD training program. Before and after the simulator training, attendees indicated on a 5-point scale the degree of difficulty in performing the following procedures: lesion marking, mucosal pre-cutting, circumferential incision, submucosal dissection, and hemostasis. After the simulator training, the participants completed a questionnaire regarding their opinions on the degree of realism and the feasibility of using this model for training.

**Results:**

After watching an instructional video, attendees felt that the most difficult techniques were submucosal dissection and hemostasis. After using the artificial tissue simulator model, the attendees felt more confident in performing performing lesion marking (*p* = 0.026) and submucosal dissection (*p* = 0.037). However, they still felt that hemostasis was the most difficult techniques to master. Overall, the attendees thought the simulator was realistic in simulated lesion marking and its use was feasible for simulated lesion marking and submucosal dissection.

**Conclusion:**

Our pilot study shows the feasibility of using a novel artificial tissue in performing ESD and we believe that the artificial tissue simulator acts well as a bridge between instructional videos and animal model training. The model is reusable and inexpensive, and could disseminate the techniques of the ESD more easily and quickly.

## Background

The techniques of therapeutic endoscopy continue to advance. Novice endoscopists require thorough training in the skills needed for endoscopic intervention [1, 2]. Since the mid-1990s, simulators have been widely used in many training programs [3, 4]. To overcome the range and depth limitations of endoscopic mucosal resection (EMR), endoscopic submucosa dissection (ESD) has been developed. ESD requires unique skills to accomplish mucosal cutting around the lesion and submucosal dissection beneath gastrointestinal neoplasms. ESD has not been adopted worldwide, partly due to the lack of a structured training system to learn the complex techniques associated with the steep learning curve separating the novice endoscopist from the proficient endoscopist [5].

It is beneficial for beginners to observe detailed videos of experts performing ESD procedures. However, simple observation of the ESD procedure does not convey the multiple skills needed for this operator-dependent technique employing varied instrumentation. A consensus statement from European experts suggested that ESD training should be performed in animal models before attempting ESD in patients [6]. Some experts have recommended trainees learn ESD initially in harvested pigs’ stomachs and subsequently in a live pig before attempting ESD in clinical practice [7]. The organ movements associated with respiration and the risks of potential bleeding or perforation during the procedure in animal models mimics the risks associated with ESD in clinical practice. However, such training must be ethically performed in compliance with institutional and national guidelines.

We believe that there is a gap between what trainees learn from an instructional video and the skills required to perform ESD in an animal model; the novice has never performed the unique and novel skills required for the ESD procedure, such as mucosal marking, mucosal pre-cutting, circumferential incision, and submucosal dissection. Training on animal models, both ex vivo or in vivo [8–10], is expensive, inconvenient, and requires time-consuming preparation. We have previously developed realistic artificial tissue models to provide a more complete ESD training experience [11]. The greatest advantage of these models is that they can be used for ESD training without the ethical issues of using live animals, and can be used multiple times for each required ESD skill. In this study, we aimed to evaluate the feasibility of using an artificial tissue model in ESD training. To our knowledge this novel approach has not been previously reported.

## Methods

### Baseline participants’ characteristics and training program

Eight GI fellows from MacKay Memorial Hospital, a medical center in Taipei with a mean age of 34 years (range, 32–35 years), and comparable academic backgrounds were enrolled in the study. They had previously undergone a didactic ESD lecture regarding the indications, proper techniques, and equipment settings for ESD. None of them had ever practiced ESD in any models or patients. Before the training program, the attendees observed the detailed techniques used during a complete ESD on video and also attended a 2-h workshop led by one certified instructor in the clinical skills training center of the MacKay Memorial Hospital. There was an initial 30 min lecture clinical ESD video, followed by the instructor demonstrating the procedures using the artificial tissue model for 30 min. The final 1 h involved hands-on practice by the fellows each. The training program was approved by the Institutional Review Board of MacKay Memorial Hospital (11MMH-ISO-024).

### The artificial tissue simulator

**Fig. 1:**
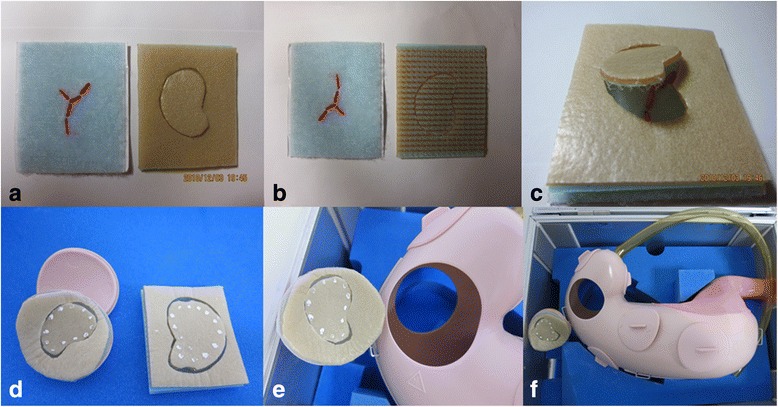
The silicon sheet is pre-cut to represent an early-stage cancer in the mucosa (**a**). The hook-and-loop fasteners consist of 2 layers of linear fabric (**b**). *Red* strands fixed on the lower layer represent exposed submucosal vessels and allow hemostasis practice (**c**). The artificial tissue is cut and mounted on a resinoid stomach model (**d** and **e**). In the resinoid stomach model, there are several ports where the artificial tissue can be mounted for ESD practice (**f**)

The artificial tissue simulator was composed of a thin flexible silicon sheet mounted on hook-and-loop fasteners (Fig. 1a). The hook-and-loop fasteners consisted of two layers of linear fabric (Fig. 1b). The upper layer consisted of small, hairy loops and represented the submucosa, while the lower layer consisted of tiny hooks and represented muscle. Red threads fixed on the lower layer represented exposed submucosal vessels (Fig. 1b). The artificial tissue was pre-cut to represent an early-stage cancer (Fig. 1c). When the 2 layers were pressed together, the hooks caught in the loops and the 2 layers temporarily bound together. The artificial tissue was cut and mounted on a resinoid stomach model (LM-083; Koken Co., Ltd., Tokyo, Japan) (Fig. 1d and e). In the resinoid stomach model, there were several port locations where the artificial tissue could be mounted for ESD practice mimicking different gastric locations (Fig. 1f).

### ESD procedures using artificial tissue

**Fig. 2:**
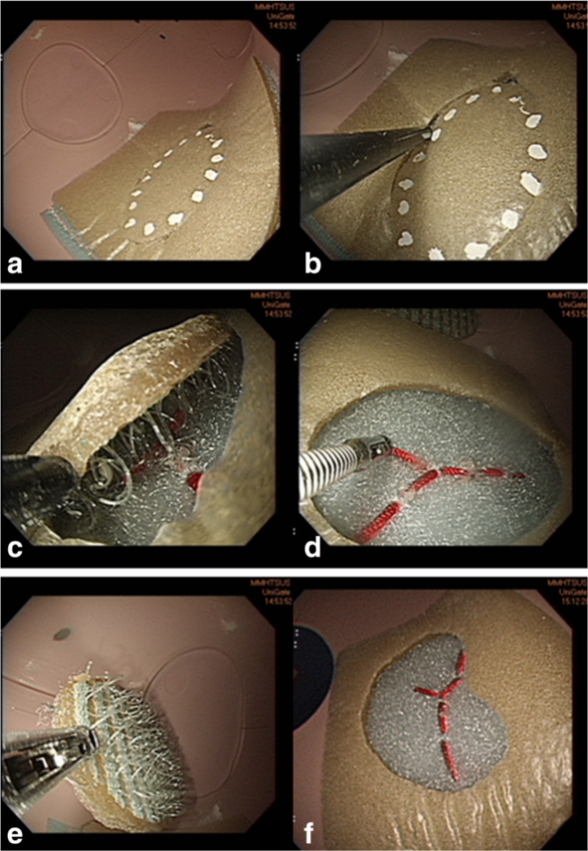
Several dots are used for marking the periphery of the lesion about 5 mm outside the edge of the target lesion. The attendees first use a dual knife to mark the periphery (**a**). The mucosa outside the markings is first cut with a dual knife after which a circumferential incision is made around the lesion (**b**). Subsequently, dissection is performed using the insulated-tip (IT-2) knife (**c**). The Coagrasper is used when visible vessels or bleeding are observed (**d**). The target lesion is then resected and removed from the model (**e**) leaving a resected mucosa (**f**)

**Fig. 3:**
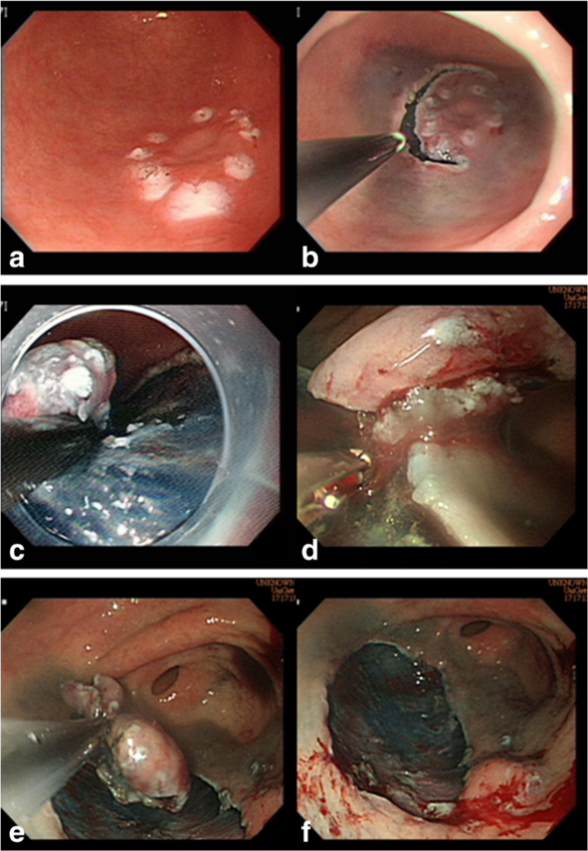
The separate manoeuvres used in ESD procedures in the clinical practice video used in this training program (**a**-**f**)

The attendees used a dual knife (KD-650Q; Olympus Optical Co., Ltd., Tokyo, Japan) to point the marker precisely for marking the periphery of the lesion for training in mucosal marking (Fig. 2a). A small initial mucosal incision (pre-cutting) was initially made necessarily deep enough to gain access to the submucosal space. A circumferential incision was then made in a direction determined by the lesion location (Fig. 2b). In ESD it is essential to maintain the dissection plane in the submucosal layer during submucosal dissection to avoid injury to the muscle layer. In our model, maintaining dissection in the proper layer avoided damage to the specimen. When the layers of the model were separated by pulling or peeling the 2 surfaces apart using an insulated-tip knife, it mimicked the process of submucosa dissection (Fig. 2c). Red threads fixed on the lower layer represented exposed submucosa vessels and helped simulate bleeding management. A Coagrasper (FD-410LR; Olympus Optical Co., Ltd., Tokyo, Japan) was used when visible vessels was identified (Fig. 2d). The target lesion was then resected and removed (Fig. 2e and f). The manoeuvres used in the simulator were similar to manoeuvres used in the standard ESD protocol employed in clinical practice (Fig. 3a-f). The attendees performed the training with a video endoscopy system, but did not use the air suction/insufflation functions or electrosurgical generator.

### Outcome measurements and statistical analysis

Before and when they practiced to familiar with the procedures in the simulator training, the attendees indicated on a 5-point scale (a score of 5 the maximum) their degree of difficulty when performing each procedure including lesion marking, mucosal pre-cutting, circumferential incision, submucosal dissection, and hemostasis. Mean score more than 4 is considered most difficult. After the simulator training, the attendees then completed a questionnaire regarding their opinions on the realism and feasibility of using this model including lesion marking, mucosal pre-cutting, circumferential incision, submucosal dissection, and hemostasis, once again using a 5-point scale (a score of 5 the maximum, mean score more than 4 is considered excellent realism and feasibility).

Given the small number of participants in this pilot study, statistical analysis of the data would be of questionable validity. These data were used for a rough comparison. For the purposes of this pilot study we used descriptive statistics, reporting the mean (± standard deviation) of the fellows’ scores. Because of the ordinal and categorical nature of the data, the Mann–Whitney *U* test was also applied to compare the fellows’ scores before and after simulator.

## Results

**Fig. 4:**
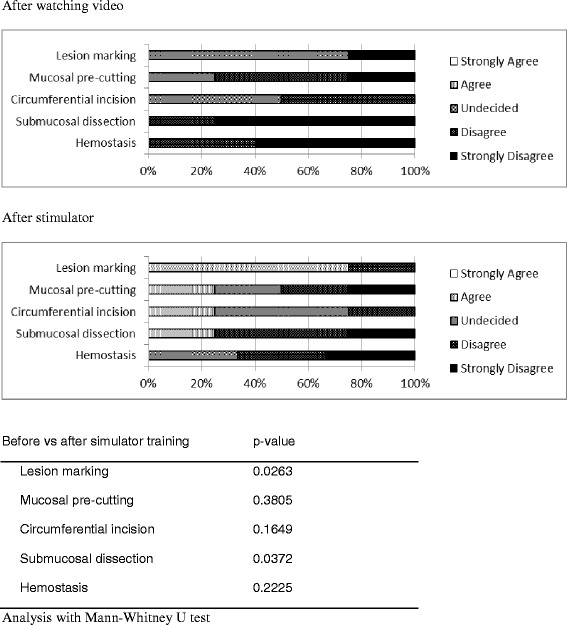
After watching video and after simulator training the fellows indicated on a 5-point scale the degree of difficulty for each procedure

After watching an instructional video, attendees felt that the most difficult techniques (mean difficulty more than 4) were submucosal dissection (4.8 ± 0.5) and hemostasis (4.8 ± 0.5) (Table 1). However, after using the artificial tissue simulator model, the attendees felt more confident in performing lesion marking (*p* = 0.026) and submucosal dissection (*p* = 0.037) and felt the techniques were easier to perform than just watching an instructional video (Fig. 4). After using the artificial tissue simulator model, they still felt the most difficult techniques (mean difficulty more than 4) to master was hemostasis (4.3 ± 1.0).

**Table 1:** After watching video and after simulator training the fellows indicated on a 5-point scale the degree of difficulty for each procedure

	After watching video	After simulator
Lesion marking	3.5 ± 1.0	2.5 ± 1.0
Mucosal pre-cutting	4.0 ± 0.8	3.5 ± 1.3
Circumferential incision	3.5 ± 0.6	3.0 ± 0.8
Submucosal dissection	4.8 ± 0.5	3.8 ± 1.3
Hemostasis	4.8 ± 0.5	4.3 ± 1.0

Descriptive statistics reporting the mean (± standard deviation) of the fellows’ scores

**Fig 5:**
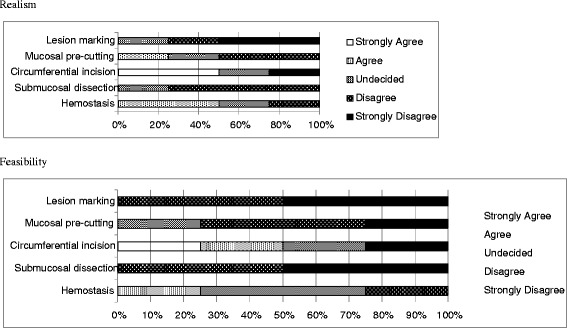
The fellows completed a questionnaire regarding their opinions on the realism and feasibility of the artificial simulator

After the workshop, the attendees completed a questionnaire regarding their opinions on the realism and feasibility of using this model. The attendees thought that simulated lesion marking (mean score 4.3 ± 1.0) was the most realistic. The attendees felt that simulator training had promising feasibility for simulated lesion marking (mean score 4.5 ± 0.6) and submucosal dissection (mean score 4.5 ± 0.6) (Table 2 and Fig. 5).

**Table 2:** The fellows completed a questionnaire regarding their opinions on the realism and feasibility of the artificial simulator

	Realism	Feasibility
Lesion marking	4.3 ± 1.0	4.5 ± 0.6
Mucosal pre-cutting	3.3 ± 1.0	4.0 ± 0.9
Circumferential incision	4.0 ± 0.9	3.8 ± 0.5
Submucosal dissection	3.8 ± 0.5	4.5 ± 0.6
Hemostasis	3.8 ± 0.5	3.0 ± 0.8

Descriptive statistics reporting the mean (± standard deviation) of the fellows’ scores

## Discussion

ESD is a complex interventional procedure involving several skills and diverse instrumentation. A satisfactory outcome for ESD requires expertise in multiple endoscopic techniques such as proper identification of the lesion, marking the periphery of the lesion, mucosal pre-cutting, submucosal dissection, and the management of bleeding and other complications. We believe that the artificial tissue simulator can be a bridge between instructional videos and animal model training. The trainees have the opportunity to practice each technique required to perform ESD and hone their skills until that point at which they are ready to attempt the animal model. The novel use of such a simulator in a training program has not been previously described.

A stepwise training process for learning ESD is widely accepted in Japan. The first step is the acquisition of basic endoscopic skills. Currently, prior to training, the novice has never performed any of the techniques necessary for the performance of ESD such as lesion marking, mucosal pre-cutting, submucosal dissection, and hemostasis of the submucosal vessels. Animal models, both ex vivo and in vivo [8–10], are expensive, inconvenient, and time consuming. The expense of hands-on training with live animals may limit the ability of some training programs to provide ESD training. One animal training session may cost nearly $300 (US) per trainee [10]. Additionally, the animal must be sacrificed afterward. The price of one ex vivo simulator with a pig stomach is $50 (US) [8]. Though the ex vivo animal models are less expensive, two hours of specimen preparation are necessary before the training session [8]. Training programs should be tailored around the program needs based on ethnicity, culture, and the working environment. With the animal model, it is possible that the novice will not be able to complete the ESD procedure in one session. Each step must be completed in sequence, and if one step is particularly challenging for the trainee, the following steps may not be completed. In this situation, use of an artificial tissue stimulator [11] (the cost is $20 (US)) at the beginning of training may save money and time, and limit the need for animal tissue. The commercial available resinoid stomach model has several port locations on which the artificial tissue can be mounted and used for multiple ESD practice sessions. The most important advantage of this novel artificial tissue model is that it provides an opportunity for inexpensive and multiple use by the novice, unlike animal models.

With practice using the artificial tissue model, the attendees felt more confident in performing each technique, and the ESD procedure became easier. It is imperative for ESD trainees to maintain a positive attitude and remain aware of their strengths and weaknesses to overcome their initial anxiety in learning the procedure. Using this training, the attendees were able to assess and improve their endoscopic manipulations. After watching an instructional video and performing ESD with the simulator, the attendees felt that the most difficult techniques were submucosal dissection and hemostasis with a mean difficulty score of 4.8. Yamamoto et al. previously reported that in a teaching program, submucosal dissection has been shown to be more difficult than mucosal incision, mostly because of uncontrolled haemorrhage [12]. The attendees in this study recognized the difficulty in performing hemostasis during ESD, and we had hoped that the red strands fixed on the lower layer, representing exposed submucosal vessels, would aid in practicing hemostasis. However, the attendees felt that in this regard the model was insufficient compared to the instructional ESD video. Obviously, expertise in hemostasis is necessary. Toeh et al. reported a 56.5 % rate of bleeding and perforation when 24 novice endoscopists used a live porcine model in an ESD training workshop in Hong Kong [13]. The attendees may gain the experiences and sufficiency training in the next step alive porcine model.

How to interpret the degree of the feasibility using this model for training? We observed the association between the decreasing difficulty and the feasibility. If the difficulty on performing each technique decreased more than one score after using the model, the attendees felt the feasibility is excellent (with a mean score more than 4) in performing such technique. For example, the attendees thought the simulator was feasible in simulated lesion marking (mean difficult score from 3.5 ± 1.0 to 2.5 ± 1.0; *p* = 0.026) and submucosal dissection (4.8 ± 0.5 to 3.8 ± 1.3; *p* = 0.037) because the techniques were easier to perform than just watching an instructional video.

Our study had a number of limitations. Because this was an in-house training project involving fellows from one institution, we had a very small sample size, precluding rigorous statistical analysis. Larger studies with an appropriate sample size and assessment of the clinical data are necessary. This pilot study provided training for almost all techniques used in ESD, but there were some differences from the use of an animal model. For instance, submucosal injections were more difficult to practice in the artificial tissue model. Novices should master their submucosal injection skills during polypectomy or EMR. Secondary, we believe that there is a long gap between learning from an instructional video and the skills required in an animal model. We did not assess performance scores in performing artificial tissue simulator in the very initial training step. It seems to help learning how to skill and proceed the multiple procedure orders employing varied instrumentation and the attendees did appear to improve confidence after the training. The third, this may raise the question of how many procedures should fellows do using artificial tissue simulator before the next step of ex-vivo animal model? When our fellows achieve success sessions in 3–5 artificial tissue simulator, they reach the learner level to performing the ex-vivo animal model. Whether those results will translate into better clinical performance on the next step animal models remains to be seen.

## Conclusion

We believe that this artificial tissue simulator should be used during the initial stages of ESD training, and should not be considered as a measure of proficiency in performing the ESD procedure clinically. It would be preferable to conduct a study on a larger scale with an appropriate sample of trainees and make recommendation. Training programs should focus on safety, efficacy, reproducibility, and cost-effectiveness regardless of conditions. Artificial tissue simulator acts well as a bridge between instructional videos and animal model training. The model is reusable and inexpensive, and should be considered for use in a stepwise training course to disseminate the techniques of the ESD procedure more easily and quickly.

## Abbreviations

EMR: Endoscopic mucosal resection
ESD: Endoscopic submucosal dissection
